# The Influence of Concreteness of Concepts on the Integration of Novel Words into the Semantic Network

**DOI:** 10.3389/fpsyg.2017.02111

**Published:** 2017-12-04

**Authors:** Jinfeng Ding, Wenjuan Liu, Yufang Yang

**Affiliations:** ^1^CAS Key Laboratory of Behavioral Science, Institute of Psychology, Beijing, China; ^2^Department of Psychology, University of Chinese Academy of Sciences, Beijing, China

**Keywords:** concreteness, novel word learning, context, semantic memory, ERP

## Abstract

On the basis of previous studies revealing a processing advantage of concrete words over abstract words, the current study aimed to further explore the influence of concreteness on the integration of novel words into semantic memory with the event related potential (ERP) technique. In the experiment during the learning phase participants read two-sentence contexts and inferred the meaning of novel words. The novel words were two-character non-words in Chinese language. Their meaning was either a concrete or abstract known concept which could be inferred from the contexts. During the testing phase participants performed a lexical decision task in which the learned novel words served as primes for either their corresponding concepts, semantically related or unrelated targets. For the concrete novel words, the semantically related words belonged to the same semantic categories with their corresponding concepts. For the abstract novel words, the semantically related words were synonyms of their corresponding concepts. The unrelated targets were real words which were concrete or abstract for the concrete or abstract novel words respectively. The ERP results showed that the corresponding concepts and the semantically related words elicited smaller N400s than the unrelated words. The N400 effect was not modulated by the concreteness of the concepts. In addition, the concrete corresponding concepts elicited a smaller late positive component (LPC) than the concrete unrelated words. This LPC effect was absent for the abstract words. The results indicate that although both concrete and abstract novel words can be acquired and linked to their related words in the semantic network after a short learning phase, the concrete novel words are learned better. Our findings support the (extended) dual coding theory and broaden our understanding of adult word learning and changes in concept organization.

## Introduction

A processing advantage of concrete concepts over abstract concepts, namely a concreteness effect, is reported in a variety of tasks including lexical decision, free recall, recognition, as well as paired associate learning (for reviews, see Paivio, 1991; Schwanenflugel et al., 1992). Concreteness effects are mainly explained by dual coding theory (Paivio, 1986) or context availability hypothesis (Schwanenflugel and Shoben, 1983).

According to the dual coding theory, there is a verbal-based system and an imagery-based system associated with concepts in semantic memory. The former is responsible for the representation and processing of linguistic information, and the latter for nonverbal information. Concrete words are connected to both the systems, while abstract words are only connected to the verbal system. When a concrete word is processed, the verbal and nonverbal systems function independently and are interconnected, which results in an additive effect, thereby yielding a processing advantage for concrete words over abstract words.

Different from the dual coding theory which emphasize that the representations of concrete and abstract words differ qualitatively, the context availability hypothesis proposes that they differ quantitively. It posits that concreteness effects arise from the differences in availability of contextual information. The contextual information can be retrieved from the person's prior knowledge or from the circumstance in which the stimulus appears. Since people encounter abstract words in a wide range of contexts or circumstances, the contextual information for abstract words is represented in a looser way. Therefore, the poor performance for abstract words is not because of lesser availability of imagery, but because of the relative unavailability of associated contextual information in memory for abstract words (Schwanenflugel et al., 1992).

Later, Holcomb et al. (1999) extended the dual coding theory with the context availability theory. They proposed that the concreteness effect could be attributed to both superior associate connections in the verbal-based system and the use of imagery-based system for concrete words. This extended dual coding theory is supported by many studies (e.g., Jessen et al., 2000; West and Holcomb, 2000; Binder et al., 2005; Zhang et al., 2006). For instance, Jessen et al. (2000) found greater activation in the lower right and left parietal lobes, as well as in the left inferior frontal lobe and in the precuneus for concrete words relative to abstract words. The stronger activation in the left parietal and frontal areas indicated greater verbal context resources for concrete words, and in the right parietal lobe indicated an additional involvement of spatial imagery-based system for concrete words.

The theories mentioned above account for the different representations between concrete and abstract words. These distinctions might reflect the way in which the words have been learned (Mestres-Missé et al., 2014). As expected, the concreteness of concepts not only impacts the processing of words, but also affects novel words learning both in L1 and L2. For instance, Palmer et al. (2013) asked native English speakers to learn rare English words (novel words) paired with definitions. Half of the novel words were concrete, and the other half abstract. It was found that participants' responses to the concrete novel words were faster than those to the abstract novel words both in semantic categorization task and lexical decision task. In L2 novel word learning, De Groot and Keijzer (2000) used paired-associate training technique, in which a Dutch word and an English pseudoword were visually presented simultaneously. Actually, the pseudowords were letter strings which were orthographically and phonologically legal. Native Dutch speakers performed recall tests immediately following learning and 1 week later. The recall accuracies for the concrete words were larger than those for the abstract words in both the tests. These results indicate that concrete words are easier to be learned than abstract words both in L1 and L2 vocabulary learning.

Contextual learning, in which people derive meanings of novel words by reading sentences or discourses, is an important approach to word acquisition (Swanborn and de Glopper, 1999; Batterink and Neville, 2011). Previous studies have found that learners can successfully infer the meaning of unknown words rapidly from contexts (Mestres-Missé et al., 2007, 2010; Borovsky et al., 2010, 2012, 2013; Chen et al., 2014; Ding et al., 2017; Zhang et al., 2017). For instance, Mestres-Missé et al. (2007) asked participants to read sentences ending in novel words (pseudowords) or real words. It was found that, only after three exposures in sentences, the N400 amplitudes elicited by the novel words were similar to those elicited by the real words. Using the same learning paradigm, researchers investigated the influence of the concreteness of concepts on novel words learning (Mestres-Missé et al., 2009, 2014). It was found that the meaning of concrete and abstract novel words could be similarly identified. However, the reading times for the abstract novel words on the second sentences were longer than those on the first sentences, and the reading times for the concrete novel words showed the reverse pattern. These results implied that participants had to collect and recheck the information provided by both sentences for abstract new words, indicating that concrete word meaning was discovered and learned faster than abstract word meaning (Mestres-Missé et al., 2014). Furthermore, an fMRI study revealed that learning concrete and abstract novel words was qualitatively different in neural correlates and recruited similar brain regions as the processing of real concrete and abstract words. In particular, the ventral anterior fusiform gyrus, a region driven by imageability (Ishai et al., 2000), was exclusively activated in the association of new concrete words to their meaning, indicating the involvement of nonverbal imagery-based system in concrete novel word leaning (Mestres-Missé et al., 2009).

The above-mentioned studies suggest a learning advantage of concrete novel words over abstract novel words during contextual learning. However, the novel words learned from contexts are not stored in isolation, they can be integrated into semantic memory rapidly (Mestres-Missé et al., 2007; Borovsky et al., 2012; Ding et al., 2017; Zhang et al., 2017). For example, Borovsky et al. (2012) found that the meaning of novel words embedded in highly constraining sentences could be learned based on the high cloze probabilities (mean = 0.896) in the pretest. Furthermore, the novel words could be associated with their semantically related words, as reflected by the reduced N400s compared to the unrelated words in a lexical decision task immediately after learning. Based on the above-mentioned results, the current study aimed to further explore whether there is a concreteness effect in the integration of novel words into semantic memory using event-related potential (ERP) technique. Specifically, we aimed to investigate how the concreteness of concepts influences the learning of novel words and their associations with known words. Most previous studies investigating word acquisition in contextual learning used pseudowords as new labels for familiar concepts (e.g., Mestres-Missé et al., 2008, 2010; Borovsky et al., 2010; Chen et al., 2014; Ding et al., 2017; Zhang et al., 2017), which can be thought of as simulating second language (L2) learning (Ferreira et al., 2015). In L2 word learning, Dittinger et al. (2016) asked speakers of French to learn Thai words through picture-word associations and found that the meaning of novel words could be learned and associated with semantically related concepts following a short learning. Furthermore, music training could enhance this kind of leaning in adults (Dittinger et al., 2016) and children (Dittinger et al., 2017). The present study paired pseudowords with familiar concepts via contextual learning paradigm and could shed new light on the influence of the concreteness of concepts on L2 novel word learning.

The novel words were Chinese two-character pseudowords and embedded in two-sentence contexts. The corresponding concepts of the novel words were either concrete or abstract. Participants read the contexts and inferred the meaning of the novel words. After learning, they performed a lexical decision task with ERPs being recorded. The learned novel words served as the primes, and the corresponding concepts of the novel words, semantically related words and unrelated words served as the targets. The N400 is a negative-going wave that peaks ~400 ms after the onset of the meaningful stimulus (Kutas and Hillyard, 1980; Kutas and Federmeier, 2011). It is correlated with semantic priming, with the target words eliciting smaller N400 amplitudes when preceded by semantically related words compared to unrelated words in the lexical decision task (Bentin et al., 1985; Estes and Jones, 2009; Borovsky et al., 2012; Jones and Golonka, 2012). Therefore, we expected smaller N400s for the corresponding concepts of the novel words and the semantically related words relative to the unrelated words in both the concrete and abstract conditions. If the concreteness of concepts influences the integration of novel words into semantic memory, the N400 effects would be different between the concrete and abstract conditions.

In addition, a late positive component (LPC) is modulated by the semantic relatedness between the prime and target, with targets preceded by semantically related primes eliciting larger LPCs than those preceded by unrelated primes in the semantic priming lexical decision task (Bouaffre and Faïta-Ainseba, 2007; Kim et al., 2012). This LPC effect reflects conscious awareness of semantic relationship between primes and targets (Bouaffre and Faïta-Ainseba, 2007; Chen et al., 2014). If the concreteness of concepts influences this late processing stage, we expected different LPC effects between the concrete and abstract conditions.

## Materials and methods

### Participants

Twenty-four university students (mean age 23 years, 12 males) participated in the experiment. They were all right handed native Chinese speakers with normal or corrected-to-normal vision. None of them had dyslexia or neural impairment. This study was approved by the Institutional Review Board of the Institute of Psychology, Chinese Academy of Sciences. Before the experiment, all subjects read and signed a written informed consent in accordance with the Declaration of Helsinki.

### Materials

Sixty-six two-character pseudowords served as the novel words. Half of the corresponding concepts of the novel words are concrete and the other half are abstract. They were embedded in the learning discourses, each of which consisted of two sentences. The first sentences always ended in the novel words. Table 1 presents the examples of stimuli. We tested the cloze probabilities of the corresponding concepts in the first sentences and the inferring probabilities of the novel words in the whole discourses. In the cloze probability test, participants read the sentences without the final critical words. They were asked to finish the sentences with the first words that came to mind. In the inferring probability test, participants read all the discourses and inferred the meaning of the novel words. Twelve participants firstly took part in the cloze probability test, then in the inferring probability test. The cloze probabilities of the concrete and abstract corresponding concepts were equally high [correct rates: mean (*SD*) = 80.8% (21.60%) and 81.06% (20.86%) for the concrete and abstract conditions, respectively: *t*_(64)_ = −0.10, *p* = 0.924]. Meanwhile, the concrete and abstract novel words could be successfully inferred at equally high accuracies [correct rates: mean (*SD*) = 96.97% (4.57%) and 96.72% (5.49%) for the concrete and abstract novel words, respectively: *t*_(64)_ = 0.20, *p* = 0.840].

Table 1Examples of the stimuli in the learning phase and lexical decision task.

**Table:** 

**LEARNING DISCOURSES IN THE LEARNING PHASE**	
**Concrete condition**	**Abstract condition**
小蝌蚪长大之后会变成一只芋沌，此刻池塘里的荷叶上蹲着一只芋沌在捕食昆虫。 (A little tadpole grows up into a **yudun**. Right now on a lotus leaf there is a **yudun** catching insects.)	获得诺贝尔奖是科学家的最高栗芸，大家都在为赢得这份最高的栗芸而努力。 (To a scientist wining the Nobel prize is the highest **liyun**. Everyone is trying hard to win this highest **liyun**.)

**Table:** 

**TARGETS IN THE LEXICAL DECISION TASK**		
	**Concrete condition**	**Abstract condition**
Corresponding concept (CC)	青蛙(frog)	荣誉(honor)
Semantically related word (SR)	蜥蜴(lizard)	声望(reputation)
Unrelated word (UR)	裤子(pants)	说法(statement)
Pseudoword	晾岌(liang ji)	贡颠(gong dian)
Pseudoword	甚筋(shen jin)	募旺(mu wang)
Pseudoword	泉愧(quan kui)	屑泊(xie bo)

*The examples are presented in Chinese with English translations in parenthesis for the learning discourses and the target words in the lexical decision task. The novel words serving as the primes are in boldface in the discourses*.

After the learning phase, the novel words served as primes in a lexical decision task paired with three types of real-word targets and three unlearned-pseudoword targets. The real-word targets included the corresponding concepts (CC), semantically related words (SR), and unrelated words (UR). For the concrete condition, the semantically related words were taxonomically/categorically related to the corresponding concepts. For the abstract condition, the semantically related words were synonyms of the corresponding concepts. Abstract words and their synonyms share semantic similarities, and their semantic relationship is similar to the categorical relationship between concrete words (Crutch and Warrington, 2005; Crutch et al., 2006). The unrelated words were concrete and abstract in the concrete and abstract conditions, respectively. Fourteen participants who did not participate in the cloze probability and inferring probability tests rated the semantic relatedness between the novel words and the semantically related words, as well as between the novel words and the unrelated words on a 7-point Likert scale (7 indicates the most closely related and 1 indicates unrelated). Since the novel words were not known before the experiment, they were replaced by the corresponding concepts. Table 2 presents the rating results. We conducted a repeated measures ANOVA, with Target condition (SR, UR) serving as a within-item factor and Word category (concrete, abstract) as a between-item factor. There was only a significant main effect of Target condition [*F*_(1, 64)_ = 3074.01, *p* < 0.001, $\eta_{p}^{2}$ = 0.980], indicating that the SR targets were more related to the novel words than the UR targets. Neither the main effect of Word category [*F*_(1, 64)_ = 2.65, *p* = 0.109, $\eta_{p}^{2}$ = 0.040] nor the interaction between Target condition and Word category [*F*_(1, 64)_ = 0.44, *p* = 0.509, $\eta_{p}^{2}$ = 0.007] was significant.

**Table 2:** Means (*SD*s) of the stimuli properties.

	**Corresponding concepts**		**Semantically related words**		**Unrelated words**	
	**Concrete**	**Abstract**	**Concrete**	**Abstract**	**Concrete**	**Abstract**
Relatedness	–	–	5.21 (0.44)	5.26 (0.51)	1.39 (0.20)	1.59 (0.27)
Concreteness	6.48 (0.39)	2.91 (0.61)	6.41 (0.48)	2.87 (0.59)	6.41 (0.28)	2.77 (0.60)
Valence	4.68 (0.73)	4.63 (0.95)	4.51 (0.83)	4.61 (0.85)	4.75 (0.70)	4.36 (0.63)
Arousal	2.80 (0.79)	3.03 (0.84)	2.61 (0.66)	2.85 (0.75)	2.62 (0.74)	2.67 (0.74)
Word frequency	2.57 (0.97)	2.52 (0.89)	2.50 (0.94)	2.36 (0.99)	2.51 (0.73)	2.34 (0.86)
Number of strokes	15.61 (4.77)	16.72 (5.10)	16.36 (4.74)	16.03 (5.14)	17.00 (3.62)	16.09 (4.08)

Another 15 participants rated all the target words in concreteness, emotional valence and arousal on 7-point Likert scales (7 indicates the most concrete, most positive, and most aroused). Meanwhile, we checked the word frequency based on the corpus developed by Cai and Brysbaert (2010) and calculated the number of strokes of all the target words. Table 2 presents the results of the ratings and calculations. We performed separate repeated measures ANOVAs for the concreteness, emotional valence, emotional arousal, word frequency, and number of strokes, with Target condition (CC, SR, UR) serving as a within-item factor and Word category (concrete, abstract) as a between-item factor. Table 3 presents *F*-values of the ANOVAs on the stimuli properties. The results showed that the target words differed in concreteness, with the concrete words being more concrete than the abstract words. In addition, all the words were matched for emotional valence and arousal, as well as word frequency and number of strokes.

**Table 3:** *F*-values of the ANOVAs on the stimuli properties.

	**Concreteness**	**Valence**	**Arousal**	**Word frequency**	**Number of strokes**
Target condition	0.76	0.40	2.54	0.48	0.14
Word category	2640.97^***^	0.73	2.06	0.62	0.95
Target condition by Word category	0.15	2.12	0.40	0.11	0.88

*The df for Target condition and the interaction was (2, 128), for Word category was (1, 64)*.

*** *Significant at 0.001 level*.

In addition, to make sure that participants learned the corresponding concepts of the novel words instead of their semantically related words since they first encountered the novel words, we calculated the cloze probabilities in the first sentences and the inferring probabilities in the whole discourses for the semantically related words. The results showed that the cloze probabilities of the concrete and abstract semantically related words were not significantly different [correct rates: mean (*SD*) = 0.51% (2.02%) and 0.25% (1.45%) for the concrete and abstract semantically related words, respectively: *t*_(64)_ = 0.83, *p* = 0.412], and were not significantly different from zero [*t*_(32)_ = 1.36, *p* = 0.184 and *t*_(32)_ = 1.00, *p* = 0.325 for the concrete and abstract conditions, respectively]. Similarly, their inferring probabilities were equally low [correct rates: mean (*SD*) = 0.76% (3.20%) and 0.25% (1.45%) for the concrete and abstract semantically related words, respectively: *t*_(64)_ = 0.58, *p* = 0.562], and were not significantly different from zero [*t*_(32)_ = 1.44, *p* = 0.160 and *t*_(32)_ = 1.00, *p* = 0.325 for the concrete and abstract conditions, respectively].

### Procedure

Participants who did not take part in any pretests of the stimuli were seated in a comfortable chair with a distance of about 80 cm from the computer screen. The words were presented in white color on a black screen with a font size of 20 in Song Typeface. Similar to the learning procedure of previous ERP studies (e.g., Zhang et al., 2017), a learning trial started with a 1,000-ms fixation cross in the center of the screen. Then a sentence was presented one word or two-word phrase at a time (500-ms duration, 800-ms stimulus onset asynchrony). The novel words always appeared in isolation for 1,000 ms. After the last phrase, the whole learning discourse was presented on the screen. Participants were asked to press the space button if they had inferred the meaning of the novel word. A 2,000-ms resting screen was presented before the next trial began.

In the lexical decision task, a trial also started with a 1,000-ms fixation cross in the center of the screen. Then a prime word was presented for 300 ms and followed by a 200-ms blank screen. After that, a target word was presented for 300 ms. Participants were asked to judge whether the target was a real word or not as quickly and accurately as possible by pressing the “F” or “J” buttons on the keyboard. The correspondence between F/J and word/pseudoword was counterbalanced across participants.

We divided the 66 discourses into six blocks. In order to balance the number of concrete and abstract items, three blocks included 10 discourses (five concrete and five abstract), and the other three blocks included 12 discourses (six concrete and six abstract). The learning phase and lexical decision task were interleaved. Participants read the discourses in a pseudo-random order in each block with no more than three discourses of the same condition being presented in succession. All word pairs in the lexical decision task were arranged in a random order first. Then, for the word pairs containing the same novel word, the novel word-CC target pair was always presented after the novel word-SR and novel word-UR target pairs. This manipulation was performed to avoid acquisition or recognition of the novel words' meaning through the pairing with their corresponding concepts, which would confound the contextual learning effect. Furthermore, no trial type occurred more than three times consecutively and trials containing the same novel words were not presented in succession. Finally, the word pairs were presented in a pseudo-random order. There was a short break between blocks.

### Electrophysiological recording and preprocessing

EEG was recorded with 64 Ag/AgCl electrodes mounted on an elastic cap at a sampling rate of 500 Hz with a band pass filter of 0.05–100 Hz. EEG data were amplified with AC amplifiers. The right mastoid electrode served as the online reference, and an electrode placed between Fz and FPz electrodes served as the ground. An electrode was also placed over the left mastoid. Two electrodes above and below the left eye were used to monitor the vertical eye movements and blinks. The horizontal eye movements were monitored via two electrodes placed lateral to the outer canthus of each eye. Impedance of most electrodes was kept below 5 kΩ.

The raw EEG data were preprocessed with NeuroScan software 4.5 offline. After automatic correction of the ocular artifacts (Semlitsch et al., 1986), the EEG data were filtered using a band-pass filter at 0.1–30 Hz and segmented into 1,200-ms epochs from −200 to 1,000 ms relative to the target words onset. The mean amplitudes in the prestimulus interval served as baseline. An artifact correction of ±80 μV was used at all electrodes except the electrooculograms. Then, the ERPs were re-referenced offline to the average of two mastoids. Finally, average ERPs were calculated for each participant at each electrode in each condition.

### ERP data analysis

**Figure 1:**
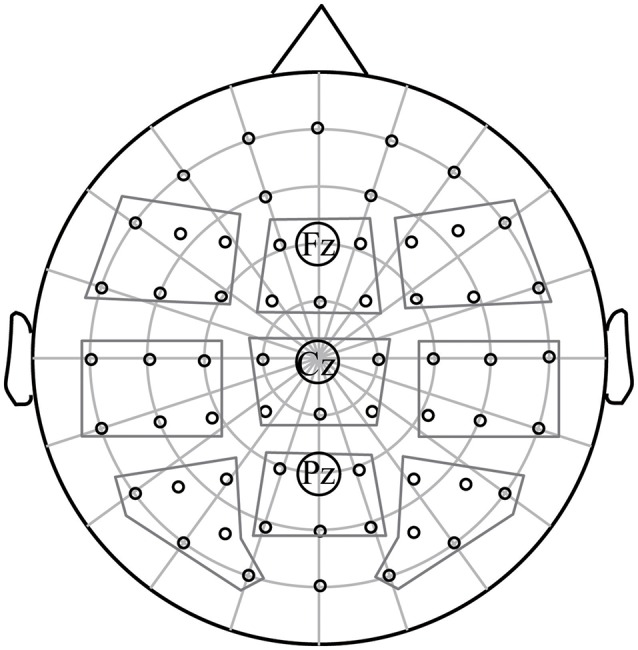
Electrode layout on the scalp. The nine regions present the electrodes selected for analysis. Electrodes Fz, Cz, and Pz were used for displaying grand average waveforms.

The mean amplitudes calculated for each participant, each condition, within each selected time window were entered into statistical analysis. Figure 1 shows the selected electrodes for analysis. Target condition (CC, SR, UR), Word category (concrete, abstract), Laterality (left, middle, right), and Anteriority (anterior, central, posterior) were taken as within-subject factors in repeated measures ANOVAs. In addition, simple effect tests and planned comparisons were conducted when there were any interactions with the critical manipulations in ANOVAs. Bonferroni correction was applied to adjust the multiple comparisons. The original degrees of freedom were reported with corrected *p*-values according to the Greenhouse-Geisser correction applied when appropriate (Greenhouse and Geisser, 1959).

## Results

### Behavioral data

**Figure 2:**
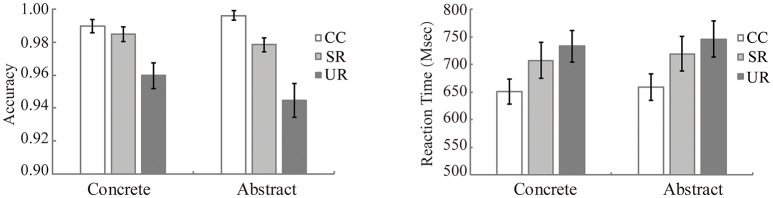
The accuracy (in percentage, **Left panel**) and the reaction time of correct responses (in ms, **Right panel**) for target words in each condition. Error bars represent the standard error. CC, corresponding concepts; SR, semantically related words; UR, unrelated words.

Figure 2 presents the accuracy results (left panel) and the reaction time results (right panel). We conducted 3 Target condition (CC, SR, UR) by 2 Word category (concrete, abstract) repeated measures ANOVAs for the accuracy and reaction time data. For accuracy, there was a significant main effect of Target condition [*F*_(2, 46)_ = 20.76, *p* < 0.001, $\eta_{p}^{2}$ = 0.474]. Pair-wise comparisons revealed that the accuracy for the CC targets was the highest [CC vs. SR: *t*_(23)_ = 3.67, *p* = 0.007; CC vs. UR: *t*_(23)_ = 5.86, *p* < 0.001]. In addition, the accuracy for the SR targets was higher than that for the UR targets [SR vs. UR: *t*_(23)_ = 3.75, *p* = 0.009]. The main effect of Word category [*F*_(1, 23)_ = 2.77, *p* = 0.110, $\eta_{p}^{2}$ = 0.107] or the interaction between Word category and Target condition [*F*_(2, 46)_ = 2.03, *p* = 0.161, $\eta_{p}^{2}$ = 0.081] was not significant.

For reaction time, error trials and outlier data points which were 2.5 standard deviations away from the mean were excluded from analysis. The repeated ANOVA revealed a significant main effect of Target condition [*F*_(2, 46)_ = 20.08, *p* < 0.001, $\eta_{p}^{2}$ = 0.466]. Pair-wise comparisons revealed that the participants responded faster for the CC targets than for the SR [CC vs. SR: *t*_(23)_ = −3.80, *p* = 0.003] and UR [CC vs. UR: *t*_(23)_ = −5.64, *p* < 0.001] targets. In addition, the responses to the SR targets were faster than those for the UR targets [SR vs. UR: *t*_(23)_ = −2.63, *p* = 0.045]. Neither the main effect of Word category [*F*_(1, 23)_ = 2.41, *p* = 0.134, $\eta_{p}^{2}$ = 0.095] nor the interaction between the two factors [*F*_(2, 46)_ = 0.15, *p* = 0.861, $\eta_{p}^{2}$ = 0.006] was significant.

### ERP data

**Figure 3:**
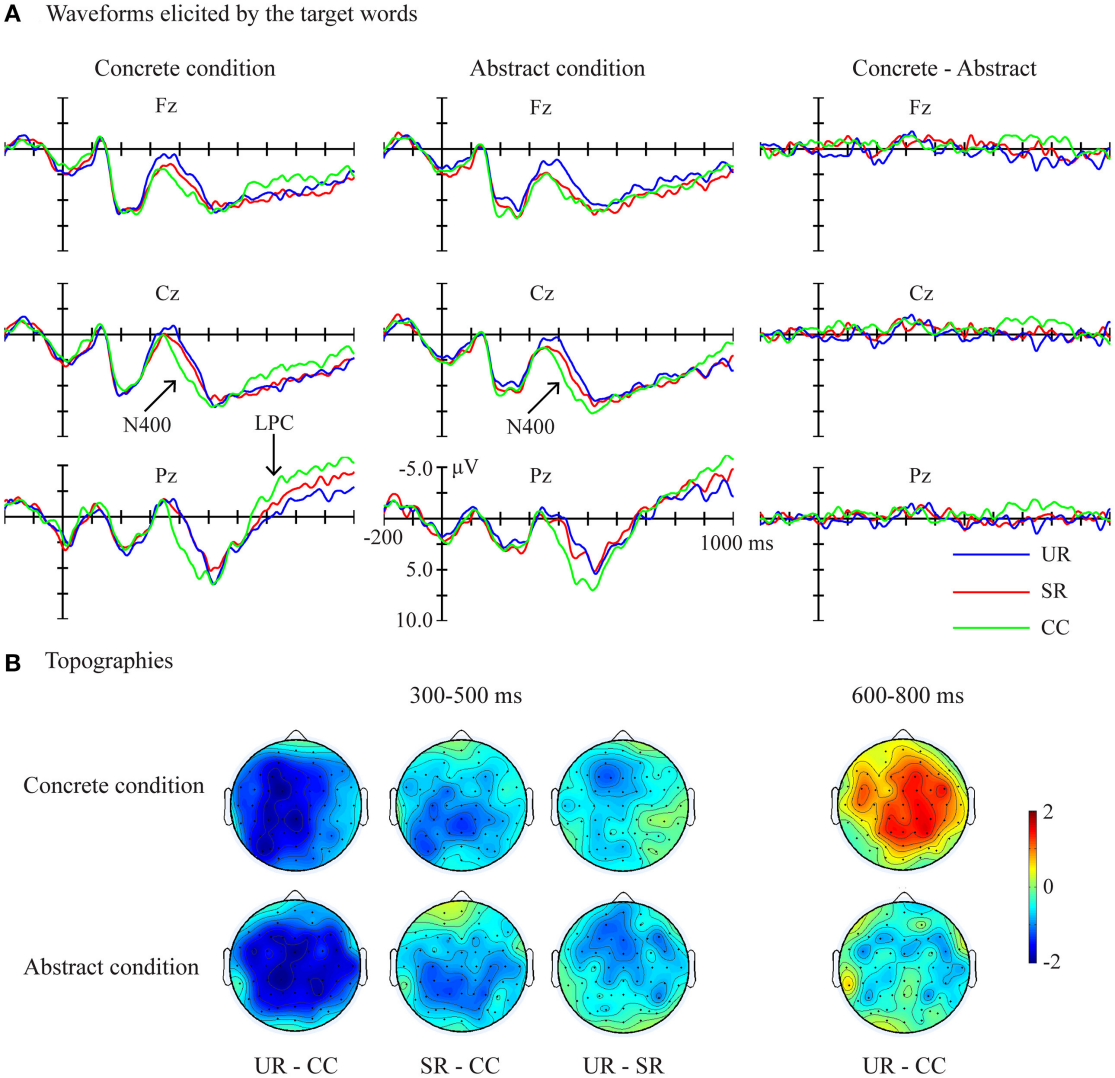
Results of the ERP analysis. **(A)** Waveforms elicited by the CC, SR, and UR targets in the concrete (Left panel) and abstract (Middle panel) conditions, as well as difference waveforms between the concrete and abstract conditions (Right panel) were presented at Fz, Cz, and Pz electrodes. **(B)** Topographies showing the average amplitude voltage differences between the CC, SR, and UR targets, respectively, in the time windows of 300–500 and 600–800 ms. CC, corresponding concepts; SR, semantically related words; UR, unrelated words.

The grand average waveforms elicited by the target words in the concrete (Left panel) and abstract (Middle panel) conditions, as well as difference waveforms between the concrete and abstract conditions (Right panel) were presented at Fz, Cz, and Pz electrodes in Figure 3. Based on visual inspection and previous studies (e.g., Zhang et al., 2017), two time windows were selected for statistical analysis: (1) the standard N400 time window: 300–500 ms; (2) the LPC time window: 600–800 ms.

The statistical analysis of the N400 component revealed a significant main effect of Target condition [*F*_(2, 46)_ = 15.88, *p* < 0.001, $\eta_{p}^{2}$ = 0.408]. Pair-wise comparisons showed that the CC targets elicited the smallest N400 amplitudes [CC vs. SR: *t*_(23)_ = 3.09, *p* = 0.015; CC vs. UR: *t*_(23)_ = 5.27, *p* < 0.001]. Meanwhile, the SR targets elicited smaller N400s than the UR targets [SR vs. UR: *t*_(23)_ = 2.71, *p* = 0.037]. The N400 effects were distributed over all electrodes tested. There were not any other significant effects.

In the 600–800 ms time window, there was a significant interaction between Target condition and Word category [*F*_(2, 46)_ = 3.78, *p* = 0.030, $\eta_{p}^{2}$ = 0.141]. Simple effect tests revealed a significant effect of Target condition in the concrete condition [*F*_(2, 46)_ = 4.30, *p* = 0.019, $\eta_{p}^{2}$ = 0.157], but not in the abstract condition [*F*_(2, 46)_ = 0.90, *p* = 0.413, $\eta_{p}^{2}$ = 0.038]. Pair-wise comparisons showed that the concrete CC targets elicited a smaller LPC than the concrete UR targets [CC vs. UR: *t*_(23)_ = −3.59, *p* = 0.005; CC vs. SR: *t*_(23)_ = −2.22, *p* = 0.109; SR vs. UR: *t*_(23)_ < 1] over all electrodes tested. No other significant main effects or interactions of interest were observed.

## Discussion

The current study aimed to examine whether and how the concreteness of concepts influences the learning of novel words and their associations with known words in semantic memory. After inferring the meaning of the novel words in the learning contexts, participants completed a lexical decision task with the learned novel words serving as the prime words. The corresponding concepts of the novel words and the semantically related words were judged faster and more accurately, and elicited smaller N400s compared to the unrelated words. The N400 effect was not modulated by the concreteness of concepts. In addition, the corresponding concepts of the concrete novel words elicited smaller LPCs than the concrete unrelated words. This LPC effect was absent in the abstract condition.

The learned novel words, irrespective of the concreteness of concepts, facilitated the processing of their corresponding concepts and semantically related words, as reflected by the behavioral data and the N400 effects. This is in line with previous studies investigating novel word acquisition in contextual learning (Mestres-Missé et al., 2007; Borovsky et al., 2012; Chen et al., 2014; Ding et al., 2017; Zhang et al., 2017). These results indicate that learners can successfully infer the meaning of novel words from highly constraining contexts and rapidly connect the novel words with their semantically related words in semantic memory.

Unlike the N400 effect, the LPC effect was modulated by the concreteness of concepts in the current study. The LPC effect has been proposed to reflect conscious awareness of semantic relationship between the prime and the target at a late processing stage (Bouaffre and Faïta-Ainseba, 2007; Chen et al., 2014), with related targets eliciting larger late positive waveforms than unrelated targets (Brown et al., 2000; Hill et al., 2002). However, the LPC effect obtained in the current study might not reflect the semantic relationship-detection (Hill et al., 2005) for two reasons. First, the LPC effect was observed for the corresponding concepts of the novel words, not for the semantically related words. Second, the corresponding concepts elicited smaller, but not larger, LPCs than the unrelated words. We propose two possibilities for the LPC effect as follows. Firstly, the LPC effect is correlated with processing difficulty (Brouwer et al., 2012); therefore, the observed LPC reduction reflected a facilitation of the concrete novel words on the processing of their corresponding concepts. In the present study, the novel words were new forms of the corresponding concepts. After learning, the novel words were stored as new labels of their corresponding concepts in semantic memory. Furthermore, due to the processing advantage of the concrete concepts, the association strength between the novel words and the concepts might be stronger for the concrete words than for the abstract words. Hence, the concrete novel words were learned more deeply than the abstract novel words in contextual reading. Secondly, the LPC has been viewed as part of the P300 family of ERP components that reflect context updating (Donchin and Coles, 1988). The larger LPCs to the unrelated words than to the corresponding concepts of the novel words in the concrete condition possibly reflected more context updating for the unrelated words, because they were less expected following the primes than the corresponding concepts. In other words, the concrete learning contexts were recollected more vividly than the abstract learning contexts and potentially more so for the concrete novel words. The process may facilitate the processing of their corresponding concepts relative to the unrelated words. This interpretation implies that episodic memory played an important role in the testing phase. It should be noted that the second possibility is not contradictory to the integration of novel words into semantic memory, as indicated by the N400 effect given its automaticity (Kutas and Federmeier, 2011).

According to the context availability hypothesis, the concreteness effect would disappear with the provision of contexts to concrete and abstract words (Schwanenflugel and Shoben, 1983; Schwanenflugel et al., 1988). For instance, the lexical decision times for the concrete and abstract words were equivalent, with a sentence providing contextual information (Schwanenflugel and Shoben, 1983). The behavioral data did not reveal a difference between the concrete and abstract conditions, which is consistent with the context availability theory. However, the context availability theory could not explain the LPC effect we observed in the concrete condition. This concreteness effect could be accounted for by the dual coding theory (Paivio, 1986) or the extended dual coding theory (Holcomb et al., 1999). The usage of the imagery-based system or more verbal information, or both of them, for the concrete concepts facilitated the learning of the concrete novel words. All these results suggest that concrete and abstract words might be quantitatively and qualitatively different. We propose, as previous studies suggested, concreteness of concepts is not a dual feature, but a continuum (e.g., Mestres-Missé et al., 2014).

It is worth to note that we also did not find a concreteness effect (i.e., main effect of Word category) in the N400 time window. Previous studied have found that concrete words elicit larger N400s than abstract words (e.g., Kounios and Holcomb, 1994; Zhang et al., 2006; Tolentino and Tokowicz, 2009; Tsai et al., 2009), reflecting more activation of semantic information from memory for concrete words (Kounios and Holcomb, 1994). This N400 effect in response to the concreteness of concepts was also observed in novel word learning studies (e.g., Palmer et al., 2013). The absence of the concreteness effect in the N400 amplitudes could be attributed to the similar context availabilities for both the concrete and abstract novel words as discussed above. However, one might argue that the concrete and abstract unrelated words might differ in context availability because they did not appear in the learning contexts. Thus, there should be a processing advantage for the unrelated words in the concrete condition over the abstract condition. We propose that the absence of the concreteness effect might be alternatively due to the relative high word frequency of the target words. Previous behavioral researches revealed no concreteness effects for high frequency words (e.g., James, 1975; De Groot, 1989; Miller and Roodenrys, 2009). The average word frequency of the critical words in the current study was 2.47, which is the log transform of the total number of times that the word appears in film subtitles (mean = 0.85, median = 0.60, mode = 0; Cai and Brysbaert, 2010). This relatively high word frequency might lead to the disappearance of the concreteness effect.

However, Zhang et al. (2006) found that concrete nouns elicited larger N400s than abstract nouns, regardless of word frequency, indicating that the concreteness effect is immune to word frequency. The different results between the current study and the study of Zhang et al. (2006) might have resulted from the discrepancies in the experimental procedure. Zhang et al. only asked participants to perform a lexical decision task, while in the current study, participants performed a lexical decision task in the semantic priming paradigm with novel words serving as primes. Hill et al. (2005) found that the N400 component was larger in the long SOA (700 ms) than in the short SOA (150 ms) for the real word targets when pseudowords served as primes. They attributed this N400 enhancement to the use of pesudoword primes which may drive the subjects to a deeper semantic processing of both the primes and targets. Therefore, the concreteness effects reflected in the N400 component might be superimposed on the deeper semantic processing. In a word, the provision of contexts and relatively high word frequency, as well as the experimental procedure might result in the absence of the concreteness effect in the behavioral data and the N400 amplitudes.

In addition, there were graded increasing N400 amplitudes for the corresponding concepts, the semantically related words and the unrelated words. These N400 priming effects were not modulated by the concreteness of concepts. First, as discussed above, these results suggest that novel words could be integrated into semantic memory rapidly, which is consistent with previous studies using contextual learning (Borovsky et al., 2012; Ding et al., 2017; Zhang et al., 2017) and picture-word associations (Dittinger et al., 2016). Second, these results are in conflict with the structurally different representational frameworks theory (Crutch and Warrington, 2005), which also proposes that the representations of concrete and abstract words differ qualitatively. Crutch and Warrington (2005) asked a patient with semantic refractory access dysphasia to perform a spoken word-written word matching task, in which the patient was required to point to the target written word in a word array following the spoken word. Words in the same array related to each other via either semantic association or semantic similarity. The results revealed interference for the semantically associated abstract words, but not for the semantically synonymous abstract words. However, the concrete words showed the reverse pattern. Based on these results, researchers proposed that the concrete concepts are organized by semantic similarity, and abstract concepts are represented in an associated neural network (Crutch and Warrington, 2005; Crutch et al., 2006). However, subsequent studies on healthy participants (Zhang et al., 2013; Geng and Schnur, 2015) and patients (Hamilton and Coslett, 2008) found that semantic similarity is also important to abstract concepts. In the current study, when preceded by the learned novel words, the synonyms of the abstract words serving as the semantically related words elicited smaller N400s than the unrelated words, indicating that the semantic similarity between concepts also plays a role in the representations of abstract words. These results again support the quantitative instead of the qualitative differences between the concrete and abstract words.

There are some limitations to this study. Firstly, the novel word-CC target pairs were always presented after the novel word-SR target and the novel word-UR target pairs. This design was to avoid the meaning of the novel words being acquired by the pairing of the novel words with their corresponding concepts. Although the design feature was equally true for the abstract and concrete words, it somewhat complicated the interpretation of the results. In future studies, more experimental stimuli and a Latin-square design for the three types of the prime-target pairs would address this issue. Secondly, the difference in the vividness between the concrete and abstract contexts, as well as the immediate test following the learning phase made it possible that episodic memory played an important role in semantic processing. Testing the integration of novel words into the semantic network at least a day after learning may partially rule out the contribution of episodic memory.

In summary, both the concrete and abstract novel words learned from contextual information could prime their corresponding concepts and the semantically related words compared to the unrelated words, as reflected by the graded increasing N400s for the three types of words. In addition, the concrete novel words impacted the processing of their corresponding concepts at a late processing stage as indicated by the LPC effect in the concrete condition. This study demonstrated that learners can infer the meaning of concrete and abstract novel words from contextual information, and integrate them into the semantic network. Furthermore, the concrete novel words are learned better than the abstract novel words. Because the word learning task in this experiment resembles second language learning, findings from this investigation shed new light on adult word learning and changes in concept organization.

## Author contributions

JD and YY: conceived the idea of the study; JD and WL: collected and analyzed the data; All authors contributed to the interpretation, drafting, critical revision, and final approval of the manuscript for publication.

### Conflict of interest statement

The authors declare that the research was conducted in the absence of any commercial or financial relationships that could be construed as a potential conflict of interest.

## References

[B1] Batterink L. Neville H. (2011). Implicit and explicit mechanisms of word learning in a narrative context: an event-related potential study. J. Cogn. Neurosci. 23, 3181–3196. 10.1162/jocn_a_00013 21452941 PMC3129368

[B2] Bentin S. McCarthy G. Wood C. C. (1985). Event-related potentials, lexical decision and semantic priming. Electroencephalogr. Clin. Neurophysiol. 60, 343–355. 10.1016/0013-4694(85)90008-2 2579801

[B3] Binder J. R. Westbury C. F. McKiernan K. A. Possing E. T. Medler D. A. (2005). Distinct brain systems for processing concrete and abstract concepts. J. Cogn. Neurosci. 17, 905–917. 10.1162/0898929054021102 16021798

[B4] Borovsky A. Elman J. L. Kutas M. (2012). Once is enough: N400 indexes semantic integration of novel word meanings from a single exposure in context. Lang. Learn. Dev. 8, 278–302. 10.1080/15475441.2011.614893 23125559 PMC3484686

[B5] Borovsky A. Kutas M. Elman J. L. (2010). Learning to use words: event-related potentials index single-shot contextual word learning. Cognition 116, 289–296. 10.1016/j.cognition.2010.05.004 20621846 PMC2904319

[B6] Borovsky A. Kutas M. Elman J. (2013). Getting it right: word learning across the hemispheres. Neuropsychologia 51, 825–837. 10.1016/j.neuropsychologia.2013.01.027 23416731 PMC3656665

[B7] Bouaffre S. Faïta-Ainseba F. (2007). Hemispheric differences in the time-course of semantic priming processes: evidence from event-related potentials (ERPs). Brain Cogn. 63, 123–135. 10.1016/j.bandc.2006.10.006 17207563

[B8] Brouwer H. Fitz H. Hoeks J. (2012). Getting real about semantic illusions: rethinking the functional role of the P600 in language comprehension. Brain Res. 1446, 127–143. 10.1016/j.brainres.2012.01.055 22361114

[B9] Brown C. M. Hagoort P. Chwilla D. J. (2000). An event-related brain potential analysis of visual word priming effects. Brain Lang. 72, 158–190. 10.1006/brln.1999.2284 10722786

[B10] Cai Q. Brysbaert M. (2010). SUBTLEX-CH: chinese word and character frequencies based on film subtitles. PLoS ONE 5:e10729. 10.1371/journal.pone.0010729 20532192 PMC2880003

[B11] Chen S. Wang L. Yang Y. (2014). Acquiring concepts and features of novel words by two types of learning: direct mapping and inference. Neuropsychologia 56, 204–218. 10.1016/j.neuropsychologia.2014.01.012 24480035

[B12] Crutch S. J. Ridha B. H. Warrington E. K. (2006). The different frameworks underlying abstract and concrete knowledge: evidence from a bilingual patient with a semantic refractory access dysphasia. Neurocase 12, 151–163. 10.1080/13554790600598832 16801151

[B13] Crutch S. J. Warrington E. K. (2005). Abstract and concrete concepts have structurally different representational frameworks. Brain 128, 615–627. 10.1093/brain/awh349 15548554

[B14] De Groot A. M. (1989). Representational aspects of word imageability and word frequency as assessed through word association. J. Exp. Psychol. Learn. Mem. Cogn. 15, 824–845. 10.1037/0278-7393.15.5.824

[B15] De Groot A. M. Keijzer R. (2000). What is hard to learn is easy to forget: the roles of word concreteness, cognate status, and word frequency in foreign-language vocabulary learning and forgetting. Lang. Learn. 50, 1–56. 10.1111/0023-8333.00110

[B16] Ding J. Chen S. Wang L. Yang Y. (2017). Thematic and taxonomic relations of novel words learned from action and perceptual features. J. Neurolinguistics 41, 70–84. 10.1016/j.jneuroling.2016.10.002

[B17] Dittinger E. Barbaroux M. D'Imperio M. Jäncke L. Elmer S. Besson M. (2016). Professional music training and novel word learning: from faster semantic encoding to longer-lasting word representations. J. Cogn. Neurosci. 28, 1584–1602. 10.1162/jocn_a_00997 27315272

[B18] Dittinger E. Chobert J. Ziegler J. C. Besson M. (2017). Fast brain plasticity during word learning in musically-trained children. Front. Hum. Neurosci. 11:233. 10.3389/fnhum.2017.00233 28553213 PMC5427084

[B19] Donchin E. Coles M. G. (1988). Is the P300 component a manifestation of context updating? Behav. Brain Sci. 11, 357–374. 10.1017/S0140525X00058027

[B20] Estes Z. Jones L. L. (2009). Integrative priming occurs rapidly and uncontrollably during lexical processing. J. Exp. Psychol. Gen. 138, 112–130. 10.1037/a0014677 19203172

[B21] Ferreira R. A. Göbel S. M. Hymers M. Ellis A. W. (2015). The neural correlates of semantic richness: evidence from an fMRI study of word learning. Brain Lang. 143, 69–80. 10.1016/j.bandl.2015.02.005 25797097

[B22] Geng J. Schnur T. T. (2015). The representation of concrete and abstract concepts: categorical versus associative relationships. J. Exp. Psychol. Learn. Mem. Cogn. 41, 22–41. 10.1037/a0037430 25068854

[B23] Greenhouse S. W. Geisser S. (1959). On methods in the analysis of profile data. Psychometrika 24, 95–112. 10.1007/BF02289823

[B24] Hamilton A. C. Coslett H. B. (2008). Refractory access disorders and the organization of concrete and abstract semantics: do they differ? Neurocase 14, 131–140. 10.1080/13554790802032218 18569737 PMC3034127

[B25] Hill H. Ott F. Weisbrod M. (2005). SOA-dependent N400 and P300 semantic priming effects using pseudoword primes and a delayed lexical decision. Int. J. Psychophysiol. 56, 209–221. 10.1016/j.ijpsycho.2004.12.004 15866325

[B26] Hill H. Strube M. Roesch-Ely D. Weisbrod M. (2002). Automatic vs. controlled processes in semantic priming - differentiation by event-related potentials. Int. J. Psychophysiol. 44, 197–218. 10.1016/S0167-8760(01)00202-1 12031295

[B27] Holcomb P. J. Kounios J. Anderson J. E. West W. C. (1999). Dual-coding, context-availability, and concreteness effects in sentence comprehension: an electrophysiological investigation. J. Exp. Psychol. Learn. Mem. Cogn. 25, 721–742. 10.1037/0278-7393.25.3.721 10368929

[B28] Ishai A. Ungerleider L. G. Haxby J. V. (2000). Distributed neural systems for the generation of visual images. Neuron 28, 979–990. 10.1016/S0896-6273(00)00168-9 11163281

[B29] James C. T. (1975). The role of semantic information in lexical decisions. J. Exp. Psychol. Hum. Percept. Perform. 1, 130–136. 10.1037/0096-1523.1.2.130

[B30] Jessen F. Heun R. Erb M. Granath D. O. Klose U. Papassotiropoulos A.. (2000). The concreteness effect: evidence for dual coding and context availability. Brain Lang. 74, 103–112. 10.1006/brln.2000.2340 10924219

[B31] Jones L. L. Golonka S. (2012). Different influences on lexical priming for integrative, thematic, and taxonomic relations. Front. Hum. Neurosci. 6:205. 10.3389/fnhum.2012.00205 22798950 PMC3394378

[B32] Kim A. S. Binns M. A. Alain C. (2012). Neuroelectric evidence for cognitive association formation: an event-related potential investigation. PLoS ONE 7:e34856. 10.1371/journal.pone.0034856 22536337 PMC3334915

[B33] Kounios J. Holcomb P. J. (1994). Concreteness effects in semantic processing: ERP evidence supporting dual-coding theory. J. Exp. Psychol. Learn. Mem. Cogn. 20, 804–823. 10.1037/0278-7393.20.4.804 8064248

[B34] Kutas M. Federmeier K. D. (2011). Thirty years and counting: finding meaning in the N400 component of the event-related brain potential (ERP). Annu. Rev. Psychol. 62, 621–647. 10.1146/annurev.psych.093008.131123 20809790 PMC4052444

[B35] Kutas M. Hillyard S. A. (1980). Event-related brain potentials to semantically inappropriate and surprisingly large words. Biol. Psychol. 11, 99–116. 10.1016/0301-0511(80)90046-0 7272388

[B36] Mestres-Missé A. Càmara E. Rodriguez-Fornells A. Rotte M. Münte T. F. (2008). Functional neuroanatomy of meaning acquisition from context. J. Cogn. Neurosci. 20, 2153–2166. 10.1162/jocn.2008.20150 18457509

[B37] Mestres-Missé A. Münte T. F. Rodriguez-Fornells A. (2009). Functional neuroanatomy of contextual acquisition of concrete and abstract words. J. Cogn. Neurosci. 21, 2154–2171. 10.1162/jocn.2008.21171 19199404

[B38] Mestres-Missé A. Münte T. F. Rodriguez-Fornells A. (2014). Mapping concrete and abstract meanings to new words using verbal contexts. Second Lang. Res. 30, 191–223. 10.1177/0267658313512668

[B39] Mestres-Missé A. Rodriguez-Fornells A. Münte T. F. (2007). Watching the brain during meaning acquisition. Cereb. Cortex 17, 1858–1866. 10.1093/cercor/bhl094 17056648

[B40] Mestres-Missé A. Rodriguez-Fornells A. Münte T. F. (2010). Neural differences in the mapping of verb and noun concepts onto novel words. Neuroimage 49, 2826–2835. 10.1016/j.neuroimage.2009.10.018 19837174

[B41] Miller L. M. Roodenrys S. (2009). The interaction of word frequency and concreteness in immediate serial recall. Mem. Cogn. 37, 850–865. 10.3758/MC.37.6.850 19679864

[B42] Paivio A. (1986). Mental Representations: A Dual Coding Approach. New York, NY: Oxford University Press.

[B43] Paivio A. (1991). Dual coding theory: retrospect and current status. Can. J. Psychol. 45, 255–287. 10.1037/h0084295

[B44] Palmer S. D. Macgregor L. J. Havelka J. (2013). Concreteness effects in single-meaning, multi-meaning and newly acquired words. Brain Res. 1538, 135–150. 10.1016/j.brainres.2013.09.015 24064384

[B45] Schwanenflugel P. J. Akin C. Luh W. M. (1992). Context availability and the recall of abstract and concrete words. Mem. Cogn. 20, 96–104. 10.3758/BF03208259 1549068

[B46] Schwanenflugel P. J. Harnishfeger K. K. Stowe R. W. (1988). Context availability and lexical decisions for abstract and concrete words. J. Mem. Lang. 27, 499–520. 10.1016/0749-596X(88)90022-8

[B47] Schwanenflugel P. J. Shoben E. J. (1983). Differential context effects in the comprehension of abstract and concrete verbal materials. J. Exp. Psychol. Learn. Mem. Cogn. 9, 82–102. 10.1037/0278-7393.9.1.82

[B48] Semlitsch H. V. Anderer P. Schuster P. Presslich O. (1986). A solution for reliable and valid reduction of ocular artifacts, applied to the P300 ERP. Psychophysiology 23, 695–703. 10.1111/j.1469-8986.1986.tb00696.x 3823345

[B49] Swanborn M. S. L. de Glopper K. (1999). Incidental word learning while reading: a meta-analysis. Rev. Educ. Res. 69, 261–285. 10.3102/00346543069003261

[B50] Tolentino L. C. Tokowicz N. (2009). Are pumpkins better than heaven? An ERP investigation of order effects in the concrete-word advantage. Brain Lang. 110, 12–22. 10.1016/j.bandl.2009.01.001 19268347

[B51] Tsai P. Yu B. H. Lee C. Y. Tzeng O. J. Hung D. L. Wu D. H. (2009). An event-related potential study of the concreteness effect between Chinese nouns and verbs. Brain Res. 1253, 149–160. 10.1016/j.brainres.2008.10.080 19059223

[B52] West W. C. Holcomb P. J. (2000). Imaginal, semantic, and surface-level processing of concrete and abstract words: an electrophysiological investigation. J. Cogn. Neurosci. 12, 1024–1037. 10.1162/08989290051137558 11177422

[B53] Zhang M. Chen S. Wang L. Yang X. Yang Y. (2017). Episodic specificity in acquiring thematic knowledge of novel words from descriptive episodes. Front. Psychol. 8:488. 10.3389/fpsyg.2017.00488 28428766 PMC5382203

[B54] Zhang Q. Guo C. Y. Ding J. H. Wang Z. Y. (2006). Concreteness effects in the processing of Chinese words. Brain Lang. 96, 59–68. 10.1016/j.bandl.2005.04.004 15913753

[B55] Zhang X. Han Z. Bi Y. (2013). Are abstract and concrete concepts organized differently? Evidence from the blocked translation paradigm. Appl. Psycholinguist. 34, 1059–1092. 10.1017/S0142716412000124

